# Effect of Intergenerational Chronic Undernutrition on Ponderal, and Linear Growth

**DOI:** 10.1155/2014/453460

**Published:** 2014-01-30

**Authors:** María Florencia Cesani, Evelia Edith Oyhenart, Héctor Mario Pucciarelli

**Affiliations:** ^1^Instituto de Genética Veterinaria, Facultad de Ciencias Veterinarias, UNLP-CONICET, CCT La Plata, La Plata 1900, Argentina; ^2^Cátedra de Antropología Biológica IV, Facultad de Ciencias Naturales y Museo, UNLP, La Plata 1900, Argentina; ^3^Departamento Científico de Antropología, Facultad de Ciencias Naturales y Museo, UNLP, La Plata 1900, Argentina

## Abstract

The aim of this paper was to analyze if intergenerational undernutrition causes growth retardation in weight and body length in two generations of rats and, if so, to assess whether the delay is cumulative. Male and female rats were assigned to one of the following groups: (1) control: they were fed ad libitum and constituted the parental generation (P), and (2) undernourished generations (F1 and F2): they were fed on 75% of the control diet. Animals were weighed and X-rayed every ten days from 20 to 100 days old in order to measure total body length. Also, body mass index was calculated. Data were processed by ANOVA and LSD post hoc tests. Impairment in weight, body length, and body mass index was found in both generations; nevertheless growth retardation was greater in F2, indicating a cumulative effect of nutritional stress. Sex differences were found, since the cumulative effect of generational undernutrition was greater and earlier in males than in females. It is concluded that when the undernutrition acts with constant intensity during several generations, the growth retardation is cumulative, indicating a negative secular trend.

## 1. Introduction

During the last century there has been, in most industrialized countries, a significant increase in body size [1–3]. These changes in growth patterns over time are known as a positive secular trend and are the result of improvements in the quality of life [4]. Conversely, some populations have experienced growth delays from one generation to the next. Authors as Komlos [5] and Bogin and Keep [6] reported that, in Europe, increased stature declined during the second half of the eighteenth century just as it did in America in the first half of the nineteenth century. Both periods were also times of rapid changes in population size, with increases in rural-urban migration, urbanization, industrialization, class division, and decline in the life expectancy of lower-income sectors. In this sense, adverse environmental factors can cause negative secular growth trend [7].

Among the environmental factors that modulate growth, nutrition plays an important role. It is known that the nutritional conditions that are experienced in early life can profoundly influence human biology and long-term health [8, 9]. Mothers who were undernourished as girls are 40 percent more likely to give birth to children who do not survive to age five. And malnourished mothers are more likely to die in childbirth. Moreover, some authors consider the undernutrition as an intergenerational factor because its effect may last beyond the generation on which it acted. The intergenerational cycle of growth failure typically described in many developing country settings is that young girls who grow poorly become stunted women and are more likely to give birth to low-birth-weight babies. If those infants are girls, they are likely to continue the cycle by being stunted in adulthood and so on [10].

The hypothesis of generational stunting on growth is a phenomenon studied in some countries [11–14]. However, given the multiplicity of factors that define the biophysical-sociocultural environment in which populations grow and develop, it is not possible to attribute the results only to nutritional factors. In this sense, experimental works that address generational issues have become very important, because they enable us to isolate the factor under study and analyze its effect on many generations, which seems impossible when studying human populations. As an example, studies like those of Stewart et al. [15], Zamenhof and van Marthens [16], Pucciarelli et al. [17], and Cesani et al. [18, 19] analyzed the effect of malnutrition on twelve, two, seven, and three generations of rats, respectively. Moreover, among researchers who have analyzed the generational effect of malnutrition there is some disagreement about the cumulative effect of this stress, possibly due to differences in the experimental design applied (i.e., kind of malnutrition, ontogenetic period in which nutritional stress acted, etc.) [19–22].

In order to enforce our knowledge about the impact of nutritional factors on growth, it is important to analyze whether intergenerational chronic undernutrition causes growth retardation in weight and body length and, if so, to assess whether the delay is cumulative. We performed a longitudinal study in two generations of rats and tested the null hypothesis which states that “when undernutrition acts with constant intensity for several generations, growth retardation will be noncumulative.”

## 2. Materials and Methods

### 2.1. Experimental Groups

Wistar rats (*Rattus norvegicus albinus*) raised at the Bioterio of the Instituto de Genética Veterinaria (IGEVET), Facultad de Ciencias Veterinarias (UNLP-CONICET, CCT La Plata), were used. The animals were kept free of pathogens and treated in compliance with standardized institutional guidelines. They were housed in solid stainless-steel cages. Room temperature ranged from 21 to 25°C, and the photoperiod consisted of 12 h of light and 12 h of dark (lights on at 06:00 h). The animals were fed on a pelleted and sterilized commercial stock diet containing proteins (25%).

When the rats reached adulthood (70 days), they were mated overnight. Pregnant rats were isolated and fed ad libitum. At birth, pups were randomly assigned to one of the following two groups:

1. control: the animals of the parental generation (P) received stock diet ad libitum from weaning (21 days old) to sampling (100 days old);
2. undernourished: pregnant rats were submitted to nutritional restriction during gestation 75% of daily food intake of a control animal of the same age (pair-feeding technique). Their offspring constituted the first filial generation (F1). Because it is well known that diet restriction during lactation substantially alters the mother's behavior, the mothers ate the stock diet ad libitum and the “overcrowding method” was adopted at this period (12 pups per litter instead of the usual eight) to ensure undernutrition. Overcrowding has been frequently employed in several studies to produce body growth retardation [19, 23]. After weaning, the animals were fed on 75% of the food eaten by their control peers. F1 adult females were mated to give birth to the second filial generation (F2). The F2 animals received the same treatment as the F1 animals.

### 2.2. Measurements

Approximately 20 males and 20 females of each generation were chosen randomly from a larger group and weighed and X-rayed every 10 days from 20 to 100 days of age in order to obtain the longitudinal data of each animal (Table 1).

Light-ether anesthesia was given during the procedure. Once the rats were sedated, they were orientated in lateral planes and radiographed using a Siemens Heliophos 4 at 240 mA/125 kV.

On each radiography the total body length (distance from rhinion to second caudal vertebra) was measured using a Fowler Max-Cal Digitrix caliper (0.01 mm accuracy). Body mass index (BMI) was calculated as body weight (g) divided by squared total body length (mm^2^).

### 2.3. Data Analysis

The goodness-of-fit for the frequency distributions was estimated by the Kolmogorov-Smirnov test for one sample. Normal distributions in all cases were found, so the data were processed by multifactor analysis of variance (ANOVA). When *F* values were significant (*P* < 0.05), post hoc comparisons were made. Post hoc analyses are usually concerned with finding patterns and/or relationships between subgroups of sampled populations that would otherwise remain undetected. To explore all possible pair-wise comparisons of means comprising a factor, we used Least Square Differences (LSD) multiple range tests.

Also, percentage differences between means (PDM) were calculated in order to obtain standardized differences between generations F1 and F2, according to the following formula [19]: PDM = 100 × (X1 − X2)/X1.

For instance, X1 = mean value of F1 and X2 = mean value of F2. If PDM (Weight) = 10, this indicates that weight in F2 is 10% less than in F1.

All statistical procedures were made with SPSS 12.0 statistical program.

## 3. Results 

ANOVA test indicated significant differences for all the factors analyzed (age, sex, and treatment), as well as for the interaction age∗sex∗treatment (Table 2).

The results of the LSD test are shown in Tables 3, 4, and 5.

*Body Weight.* The comparisons between P-F1 and P-F2 indicated significant differences and positive values from 30 days in both sexes. F1 versus F2 showed significant differences and positive values from 60 days in males and 80 days in females (Table 3).

*Body Length*. The comparisons between P-F1 and P-F2 indicated significant differences and positive values at all the ages analyzed and in both sexes. In males, F1 versus F2 showed significant differences with negative values at 20–30 days and positive values from 80 days. On the other hand, in females there were significant differences with negative values at 30–50 days and positive values at 100 days of age (Table 4).

*Body Mass Index*. In males, the comparisons between P-F1 and P-F2 indicated significant differences and positive values from 40 and 30 days of age, respectively. In females there were significant differences (positive values) from 30 (P-F1) and 40 (P-F2) days of age. F1 versus F2 showed significant differences with positive values at 20 and 50–90 days of age and negative values at 30 days in males and also significant differences with negative values at 30 days and positive values at 40, 50, 80, and 100 days of age in females (Table 5).

## 4. Discussion

Both control and undernourished animals ponderal and linear growth increased with age. However, the weight and body length average reached at 100 days of age was lower in undernourished animals, indicating growth retardation. In P generation males, the weight gain was 213 g, whereas F1 and F2 animals showed a weight reduction of 24% and 29%, respectively. Females showed a similar pattern, but the average values were lower than in males (a decrease of P: 148.6 g; F1: 22% and F2: 24%).

**Figure 1:**
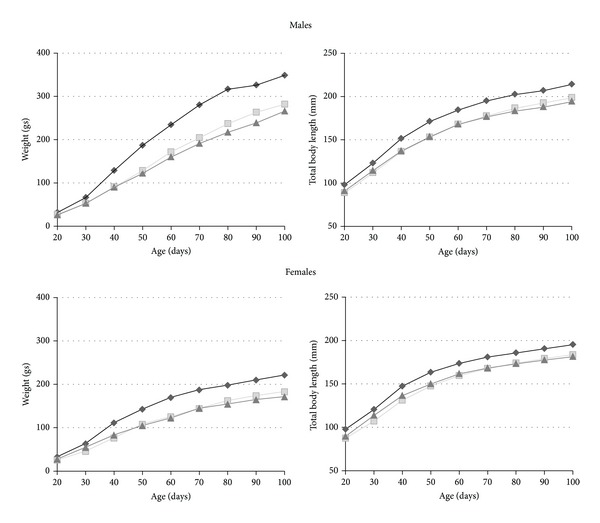
Average growth curves of weight and length in males and females. Generation P: black line; generation F1: gray line; generation F2: dark gray line.

Nutritional deficit also modified longitudinal growth but to a lesser extent than weight (males: 172.1 mm, F1: 8.6% and F2: 9.1%, and females: P: 161.8 mm, F1: 8.1% and F2: 7.1%) (Figure 1). These differential growth patterns resulted in modifications of BMI in both filial generations. Accordingly, Stewart et al. [15] reported that weight was more affected than body length in twelve generations of undernourished rats. However, even when both F1 and F2 showed impairment in weight and body length, the growth delay was more evident in the second generation, showing an intergenerational cumulative effect of nutritional stress. Accordingly, some authors support a progressive impairment of growth through generations. For example, Resnick and Morgane [24] found that a protein restriction in the first generation becomes more severe in the second one, based on brain weight at birth among other parameters. In contrast, Zamenhof and van Marthens [16] argue against this idea, because they did not find cumulative effects on growth in six generations of malnourished rats. On the other hand, Liang et al. [25] reported a reduction of body weight in the first generation of rats with a gestational protein-calorie restriction (70%) and postnatal normal nutrition but not in F2. These results evidence that postnatal nutritional rehabilitation buffers the generational effect of undernutrition. However, when nutritional stress becomes chronic during all ontogeny, it produces cumulative growth retardation.

**Figure 2:**
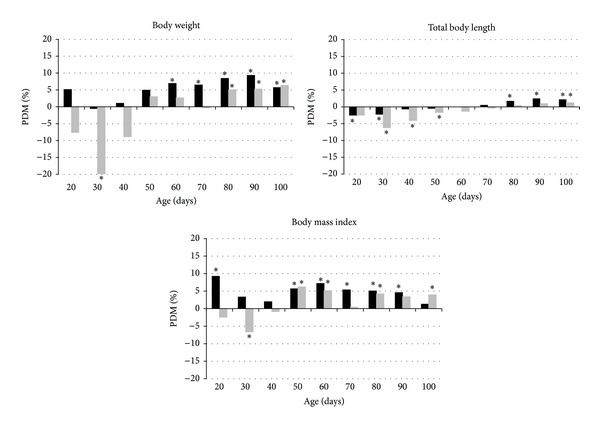
Percent differences between means (PDM) of F1 and F2 in weight, total body length, and body mass index in males (black bars) and females (gray). Positive PDMs indicate that F2 < F1. *Significant differences (*P* < 0.05).

This cumulative effect was seen in males' weight from 60 days and females' weight from 80 days, while in body length it was noted from 80 and 100 days of age in males and females, respectively. Also, the BMI was smaller in the second generation from 50 days, resulting in changes in the allometric growth (Figure 2). According to Roberts et al. [26], adult organisms have a reduced ability to “respond” to a nutritional stress. However, even when the energy demand is higher at early life, younger animals were more effective in achieving homeostasis, because the cumulative effect of generational malnutrition was evident in adulthood. These results demonstrate the importance of longitudinal studies, since if growth had been analyzed only at birth or at weaning we had assumed that there was no cumulative intergenerational effect.

Also, it is interesting to note that both the growth retardation observed in F1 and F2 and the intergenerational cumulative effect of undernutrition were greater and earlier in males than in females. These results are consistent with those reported by Stewart and Sheppard [27] and Stewart et al. [15] in twelve generations of rats fed on a protein-calorie deficient diet. It is known that under certain nutritional conditions and even when both sexes are subject to the same kind and intensity of stress females show greater capacity to keep homeostasis [28, 29]. In Tanner's terminology, female growth is “better canalized,” probably due to specific adaptive mechanisms related to their role in the reproduction process [30].

These results showed that the nutritional stress may influence the trajectories of long-term growth of rats and results in later life. Nevertheless, how these changes could be cumulative into the next generation needs an explanation. Some evidence indicates that epigenetic events mediate developmental plasticity and that chromatin modifications may be transmitted intergenerationally to influence the development of subsequent generations, especially when they are acquired during development and transition between life-history phases [31]. In this regard, new studies on the epigenetics field may help to understand this phenomenon.

## 5. Conclusions

Intergenerational undernutrition produces cumulative ponderal and linear growth retardation in both males and females, which is more evident in adulthood. Weight is more affected than body length, resulting in a change in the allometric growth. Also, it modifies the patterns of sexual differences, inasmuch as cumulative effect of generational undernutrition is greater and earlier in males than in females.

It is concluded that when the undernutrition acts with constant intensity during several generations, the growth retardation is cumulative. Intergenerational nutritional studies enable us to analyze specific adaptive processes and, therefore, the evolution of human populations. Although these experimental results cannot be directly extrapolated to humans, they allow us to advance knowledge of the negative secular trend of human populations.

## Figures and Tables

**Table 1:** Samples and treatments.

Generation	Treatment	Males	Females	Total
Parental (P)	Normal nutrition (control)	20	21	41
First filial (F1)	Intergenerational undernutrition	22	20	42
Second filial (F2)	Intergenerational undernutrition	20	22	42
Total		**62**	**63**	**125**

**Table 2:** Multifactor analysis of variance (ANOVA) for generation, sex, age, and interaction between factors.

Variable	Factor							
	Generation		Sex		Age		Interaction	
	*F*	*P*	*F*	*P*	*F*	*P*	*F*	*P*
Weight	1823.11	∗∗	5024.16	∗∗	5396.88	∗∗	8.32	∗∗
Total body length	1902.77	∗∗	1594.83	∗∗	12682.53	∗∗	2.01	∗
Body mass index	462.23	∗∗	2596.46	∗∗	1353.44	∗∗	2.85	∗

*P < 0.05.

**P < 0.01.

**Table 3:** Multiple range tests (LSD) for generational differences in weight.

	Age (days)																	
	20		30		40		50		60		70		80		90		100	
	MD	*P*	MD	*P*	MD	*P*	MD	*P*	MD	*P*	MD	*P*	MD	*P*	MD	*P*	MD	*P*
Males																		
P-F1	4.4		13.76	∗∗	37.9	∗∗	58.36	∗∗	62.65	∗∗	75.92	∗∗	79.72	∗∗	62.99	∗∗	66.68	∗∗
P-F2	5.8		13.5	∗∗	38.85	∗∗	64.7	∗∗	74.55	∗∗	89.2	∗∗	99.7	∗∗	87.5	∗∗	82.8	∗∗
F1-F2	1.41		−0.26		0.95		6.33		11.91	∗∗	13.28	∗∗	19.98	∗∗	24.5	∗∗	16.12	∗∗

Females																		
P-F1	7.33		17.8	∗∗	34.95	∗∗	34.73	∗∗	44.04	∗∗	43.01	∗∗	35.48	∗∗	36.13	∗∗	38.36	∗∗
P-F2	5.43		8.82	∗	28.23	∗∗	37.97	∗∗	47.37	∗∗	42.54	∗∗	43.66	∗∗	45.29	∗∗	49.95	∗∗
F1-F2	−1.91		−8.98	∗	−6.73		3.24		3.33		−0.47		8.18	∗	9.17	∗	11.59	∗∗

MD: mean difference.

*P < 0.05.

**P < 0.01.

**Table 4:** Multiple range tests (LSD) for generational differences in total body length.

	Age (days)																	
	20		30		40		50		60		70		80		90		100	
	MD	*P*	MD	*P*	MD	*P*	MD	*P*	MD	*P*	MD	*P*	MD	*P*	MD	*P*	MD	*P*
Males																		
P-F1	9.27	∗∗	11.15	∗∗	15.12	∗∗	18.39	∗∗	16.68	∗∗	17.46	∗∗	15.87	∗∗	14.21	∗∗	15.44	∗∗
P-F2	7.08	∗∗	8.59	∗∗	14.29	∗∗	17.7	∗∗	16.52	∗∗	18.5	∗∗	19.19	∗∗	19.00	∗∗	19.91	∗∗
F1-F2	−2.2	∗	−2.57	∗	−0.83		−0.69		−0.16		1.04		3.32	∗∗	4.80	∗∗	4.46	∗∗

Females																		
P-F1	10.33	∗∗	13.45	∗∗	16.16	∗∗	15.9	∗∗	13.99	∗∗	13.31	∗∗	11.74	∗∗	11.34	∗∗	11.59	∗∗
P-F2	8.17	∗∗	6.82	∗∗	10.86	∗∗	13.42	∗∗	11.89	∗∗	12.67	∗∗	12.55	∗∗	13.03	∗∗	13.85	∗∗
F1-F2	−2.16		−6.63	∗∗	−5.31	∗∗	−2.48	∗	−2.1		−0.64		0.80		1.69		2.26	∗

MD: mean difference.

*P < 0.05.

**P < 0.01.

**Table 5:** Multiple range tests (LSD) for generational differences in body mass index.

	Age (days)																	
	20		30		40		50		60		70		80		90		100	
	MD	*P*	MD	*P*	MD	*P*	MD	*P*	MD	*P*	MD	*P*	MD	*P*	MD	*P*	MD	*P*
Males																		
P-F1	−0.02		0.02		0.07	∗∗	0.09	∗∗	0.08	∗∗	0.09	∗∗	0.09	∗∗	0.05	∗∗	0.05	∗∗
P-F2	0.01		0.03	∗∗	0.08	∗∗	0.12	∗∗	0.12	∗∗	0.12	∗∗	0.13	∗∗	0.08	∗∗	0.06	∗∗
F1-F2	0.03	∗∗	0.01		0.01		0.03	∗∗	0.04	∗∗	0.03	∗∗	0.03	∗∗	0.03	∗∗	0.01	

Females																		
P-F1	0.01		0.04	∗∗	0.07	∗∗	0.04	∗∗	0.07	∗∗	0.06	∗∗	0.04	∗∗	0.04	∗∗	0.04	∗∗
P-F2	0.00		0.01		0.07	∗∗	0.07	∗∗	0.10	∗∗	0.06	∗∗	0.06	∗∗	0.06	∗∗	0.06	∗∗
F1-F2	−0.01		−0.03	∗∗	0.00		0.03	∗∗	0.03	∗∗	0.00		0.02	∗	0.02		0.02	∗

MD: mean difference.

*P < 0.05.

**P < 0.01.
